# Autonomic Dysfunction and Management after Spinal Cord Injury: A Narrative Review

**DOI:** 10.3390/jpm12071110

**Published:** 2022-07-07

**Authors:** Austin M. Henke, Zackery J. Billington, David R. Gater

**Affiliations:** 1 Department of Physical Medicine and Rehabilitation, University of Miami Miller School of Medicine, Miami, FL 33136, USA; austin.henke@jhsmiami.org (A.M.H.); zackery.billington@jhsmiami.org (Z.J.B.); 2 Christine E. Lynn Rehabilitation Center for the Miami Project to Cure Paralysis, Miami, FL 33136, USA; 3 The Miami Project to Cure Paralysis, University of Miami Miller School of Medicine, Miami, FL 33136, USA

**Keywords:** spinal cord injury, tetraplegia, paraplegia, autonomic dysfunction, autonomic dysreflexia, orthostatic hypotension, thermoregulatory dysfunction

## Abstract

The autonomic nervous system (ANS), composed of the sympathetic and parasympathetic nervous systems, acts to maintain homeostasis in the body through autonomic influences on the smooth muscle, cardiac muscles, blood vessels, glands and organs of the body. The parasympathetic nervous system interacts via the cranial and sacral segments of the central nervous system, and the sympathetic nervous system arises from the T1–L2 spinal cord segments. After a spinal cord injury (SCI), supraspinal influence on the ANS is disrupted, leading to sympathetic blunting and parasympathetic dominance resulting in cardiac dysrhythmias, systemic hypotension, bronchoconstriction, copious respiratory secretions and uncontrolled bowel, bladder, and sexual dysfunction. Further, afferent signals to the sympathetic cord elicit unabated reflex sympathetic outflow in response to noxious stimuli below the level of SCI. This article outlines the pathophysiology of SCI on the ANS, clinical ramifications of autonomic dysfunction, and the potential long-term sequelae of these influences following SCI.

## 1. Introduction

The autonomic nervous system (ANS) regulates visceral functions and maintains homeostasis within the human body, and while integrated with the central nervous system (CNS), it can function autonomously. Traditionally, the ANS is subdivided into the sympathetic nervous system (SNS) and parasympathetic nervous system (PNS) which are in constant opposition to each other in order to maintain appropriate homeostasis and respond to whole body perturbations [1,2]. Additionally, the gut is comprised of the enteric nervous system (ENS), an intrinsic neural network which can function independently from digest and eliminate foodstuffs, although it is usually under the influence of SNS, PNS and somatic nervous system influences [3,4]. Spinal cord injury (SCI) influences all three components of the ANS due to their relative anatomical locations, loss of supraspinal influence and unabated responses to afferent stimuli, resulting in pathophysiological responses in the cardiovascular, thermoregulatory, respiratory, gastrointestinal, and genitourinary systems that contribute to the comorbidities and mortality of SCI [2,5].

## 2. Sympathetic Nervous System

**Figure 1:**
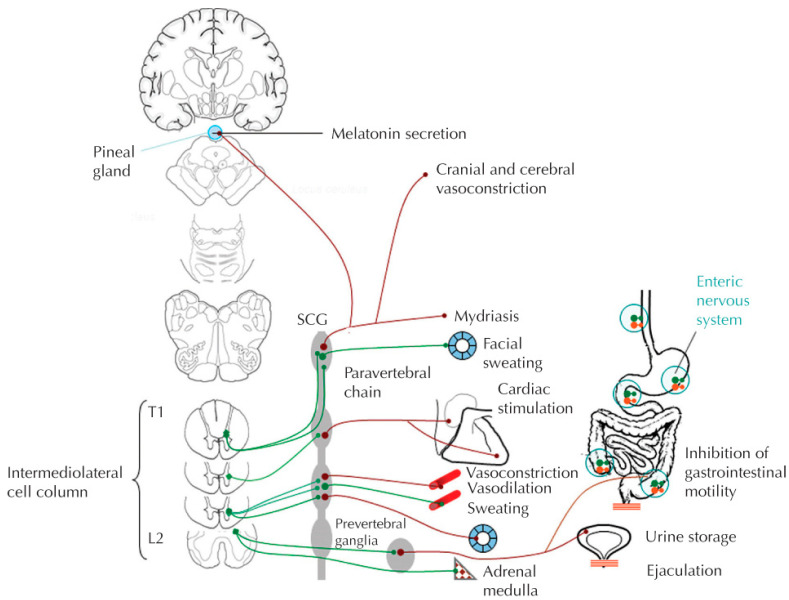
Main effectors and effects of the sympathetic system. The effects of the sympathetic system are mediated by neurons of the paravertebral and prevertebral ganglia and by the adrenal medulla. SCG, superior cervical ganglion. With permission from E.E. Benarroch. Autonomic Neurology. Oxford University Press. NY, NY. © Mayo Foundation for Medical Education and Research 2014.

The sympathetic nervous system (SNS) arises from the thoracolumbar regions of the spinal cord, with preganglionic cell bodies located in the intermediolateral horns of the spinal cord. Visceral and somatic afferent neurons with cell bodies in the segmental dorsal root ganglion synapse upon the preganglionic cell bodies directly and through interneurons, mediating reflex sympathetic responses that are usually inhibited/modified by supraspinal influences from the anterior and midcingulate cortex, insular cortex, amygdala, hypothalamus, and medulla [1]. Axons of the preganglionic sympathetic neurons exit the spinal cord through ventral nerve roots which they leave through white ramus communicans and synapse on post-ganglionic neurons located in paravertebral (sympathetic chain), prevertebral (celiac, superior, and inferior mesenteric) or terminal ganglia, as well as the adrenal medulla. (Figure 1) Preganglionic sympathetic fibers activate postganglionic sympathetic fibers through the neurotransmitter acetylcholine. They may also ascend or descend the sympathetic chain ganglion trunk to synapse on postganglionic fibers above or below their segmental exit. Paravertebral ganglia innervate all tissues and organs except those in the abdomen, pelvis, and perineum, whereas prevertebral ganglia innervate the viscera and blood vessels of the abdomen and pelvis. Postganglionic sympathetic neurons exit their respective ganglia through gray ramus communicans where they join efferent nerve roots to their effector organs, releasing norepinephrine as their primary neurotransmitter. Two notable exceptions include those postsynaptic sympathetic fibers innervating sweat glands that release acetylcholine, and the adrenal medulla that secretes epinephrine from chromaffin cells directly into the venous system [1]. Epinephrine activates α adrenergic receptors that usually serve to increase cell function (except for the gastrointestinal tract) and β adrenergic receptors that usually decrease cell function (except the heart). α1 receptors are post-synaptic and mediate vasoconstriction, whereas α_2_ receptors are presynaptic and further regulate the release of norepinephrine. Activation of β_1_ receptors increase heart rate and contractility, whereas β_2_ receptors that are plentiful in skeletal muscle but rare in renal capillary beds cause vasodilation and bronchodilation. Norepinephrine has no “R” (methyl) group on its amine terminus, so it mimics epinephrine with regard to receptor activation except for β_2_ receptors, which it cannot activate. Sympathetic signals to organs are regionalized to different spinal segments; the heart is innervated by T1–5 segments, the gastrointestinal (GI) tract is innervated by T6–11 segments, the kidneys are innervated by T10–12 segments, and the lower urinary tract and reproductive organs are innervated by T10–L2 levels. Sympathetic influence on arterial and venous vasculature is mediated by T1–5 segments for upper extremities and T6–L2 segments for the lower extremities.

Crisis situations that warrant “fight or flight” responses cause withdrawal of parasympathetic influence and activate the SNS through supraspinal “central drive” structures listed above, resulting in adrenergic outflow from postganglionic sympathetic neurons that dilate the pupils, inhibit salivation, stimulate heart rate and contractility, dilate bronchioles, activate sweat glands, constrict blood vessels in the skin, splanchnic bed and nonworking muscles, inhibit bladder and bowel contractions and contract bladder neck and internal sphincters. Of note, with intact nervous systems, the SNS always maintains some sympathetic tone, even when the body is at rest.

## 3. Parasympathetic Nervous System (PNS)

**Figure 2:**
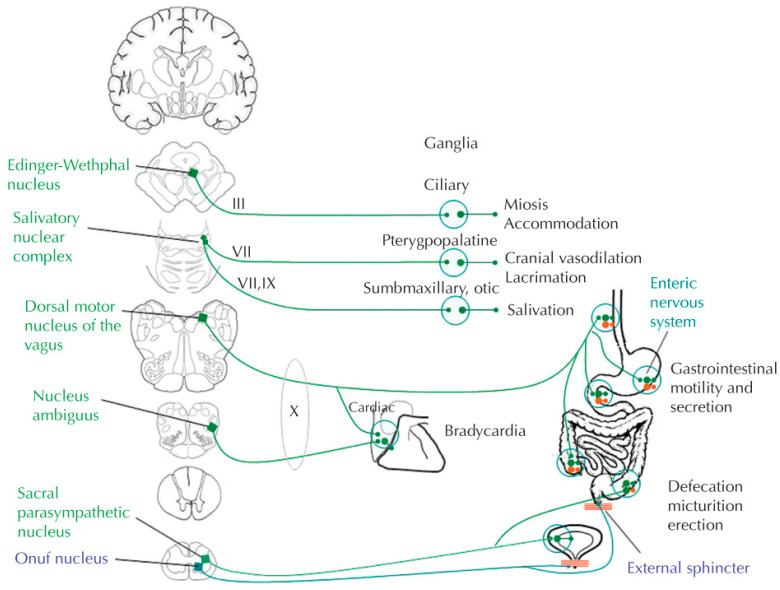
Main effectors and effects of the parasympathetic system. The effects of the parasympathetic system are mediated by neurons located in the vicinity or within the wall of the effector structures. With permission from E.E. Benarroch. Autonomic Neurology. Oxford University Press. NY, NY. © Mayo Foundation for Medical Education and Research 2014.

Preganglionic cell bodies of the PNS arise from the craniosacral axis, with the oculomotor (CN III) nerve activating pupillary constriction and lens accommodation via the ciliary ganglion, the facial nerve (CN VII) influencing lacrimation and salivation via submaxillary and submandibular ganglia, the glossopharyngeal (CN IX) nerve activating parotid gland salivation via the otic ganglion, the vagus (CN X) nerve primarily influencing thoracic and abdominal viscera, while the S1, S2 and S3 sacral nerves provide parasympathetic output to the bladder, rectum, and sexual organs [1]. (Figure 2) As opposed to the sympathetic nervous system, preganglionic efferent fibers from the parasympathetic nervous system tend to be relatively long, whereas post-ganglionic fibers to effector organs are short and secrete acetylcholine as their primary neurotransmitter, rather than norepinephrine. Target organ responses to post-ganglionic parasympathetic neurons’ release of acetylcholine are mediated through muscarinic (M) receptors, including excitatory M_1_ and M_3_ cholinergic receptors, and inhibitory cholinergic M_2_ receptors. Most of the excitatory effects of the PNS are mediated through the M_3_ cholinergic receptors, including intestinal and bladder smooth muscle contraction, exocrine gland excretion and endothelial synthesis of nitric oxide. M_3_ receptors also mediate sweat gland activation. M_1_ receptors in the stomach similarly respond by increasing gastric acid secretions when activated. Conversely, M_2_ cholinergic receptors mediate the inhibitory effects of the vagus nerve on the sinoatrial and atrioventricular nodes in the heart.

Following an SNS “crisis-like” event, sympathetic drive is withdrawn and the PNS is activated by supraspinal structures in the midbrain and hypothalamus to restore homeostasis by repairing, replacing, and replenishing any substrates that had been utilized during the sympathetically mediated event. Parasympathetic control of the cardiovascular and upper gastrointestinal (GI) tract is mediated through the vagus nerve, whereas sacral (S2–S4) elements mediate parasympathetic influence on the lower GI tract, bladder and reproductive organs. Of note, the right vagus nerve exerts more control over the sinoatrial node mediating bradycardia, whereas the left vagus nerve has greater influence on the atrioventricular node, likely leading to cardiac arrhythmias under conditions of sympathetic blunting [6]. As with the SNS, the PNS is constantly active even during crisis situations, when it withdraws most but not all of its “tone.” In fact, under normal physiological conditions, SNS and PNS are in a constant “tug-of-war,” with neither system relinquishing all of its influence even under “pure fight/flight” or pure “rest and digest” circumstances.

Disruption to these systems by the complex pathophysiology associated with SCI can cause clinically significant autonomic dysreflexia, cardiac dysrhythmias, persistent neurogenic orthostatic hypotension, thermoregulation dysfunction, respiratory dysfunction, immune dysfunction, neurogenic bowel and bladder, as well as sexual dysfunction [1,2].

## 4. Mechanism of SCI: Emerging from Neurogenic Shock

Spinal cord injury primarily damages the astrocytes, neurons, microglia, and oligodendrocytes, disrupting the neural parenchyma and axonal networks of the spinal cord [3]. Secondary injury results from swelling, inflammation, reactive oxygen species and release of excitatory amino acids that inhibit neuronal repair and growth. Acutely, the somatic and autonomic systems are in a state of neurogenic shock, with areflexia and profound unopposed parasympathetic dominance due to sympathetic blunting in SCI above T6, resulting in neurogenic bradycardia, neurogenic orthostatic hypotension (NOH), bronchiolar constriction, mucus secretion and priapism accompanying the sensory and motor paralysis associated with traumatic SCI. Additionally, those with traumatic SCI often have hypovolemic shock associated with blood loss due to internal injuries that should be accompanied by compensatory tachycardia but is offset by the parasympathetic dominance. The acute neurogenic bradycardia associated with high thoracic and cervical SCI may require transcutaneous pacing acutely in order to maintain appropriate cardiac output. It is important for the clinical team to recognize both types of shock to judiciously provide fluid resuscitation and pressor support without putting the individual into fluid overload and pulmonary hypertension. Mean arterial pressure (MAP) is determined as systolic blood pressure (SBP) + [2 × Diastolic Blood Pressure (DBP)]/3. During the acute stage of SCI, it is recommended to maintain mean arterial pressure (MAP) of 85–90 mm hg for seven days by IV fluid resuscitation, inotropes and chronotropes as needed [2,5,7] Chronically, persons with high thoracic and cervical SCI may stabilize with baseline sitting SBP in the 90–110 mmHg range.

## 5. SCI Influence on ANS Dysfunction

### 5.1. Cardiovascular Dysfunction

As above, SNS blunting due to cervical and high thoracic SCI has profound influence on cardiovascular physiology acutely, and although somewhat mitigated in chronic SCI by progressive partial PNS withdrawal over time, parasympathetic dominance and blunted supraspinal sympathetic drive continues to dampen “crisis” responses chronically. Unopposed vagal influence on the cardiac SA and AV nodes results in neurogenic bradycardia and risk of AV block; even under maximal exercise influence, peak heart rates seldom exceed 120 beats per minute (bpm). Heart rate variability (HRV) has been assessed with spectral analysis in cervical and high thoracic SCI demonstrating parasympathetic dominance with increased high frequency (HF) domains; variable low frequency (LF) domains may reflect sympathetic or a combination of sympathetic and parasympathetic activity [8,9,10,11]. While HRV may provide some indication of relative SNS:PNS activity following acute SCI [11], it has proven to be a less reliable indicator in chronic SCI and warrants further investigation [9]. Blunted SNS results in vasodilation in the viscera and extremities, as vascular activation of α_1_ receptors is blocked and systemic vasoconstriction to maintain blood pressure/tissue perfusion is markedly diminished, resulting in NOH [12]. NOH is clinically defined as a sustained blood pressure fall ≥ 20 mmHg systolic (SBP) and/or fall ≥ 10 mmHg diastolic (DBP) within 3 min of active standing or sitting upright from a supine position [12]. Illman et al. found that 74% of SCI patients suffer from orthostatic hypotension and 59% of those patients had symptomatology that prevents physiotherapy sessions while in inpatient rehabilitation [13]. Venous vascular tone is also severely diminished with SNS blunting, and in conjunction with absent/diminished lower extremity muscle contraction due to paralysis, venous pooling/gravity-dependent lower extremity edema ensues, and inferior vena cava dilation occurs. Subsequently, left ventricular end diastolic volume is markedly diminished, reducing preload and myocardial contractility according to the Starling mechanism. With both afterload and preload diminished, adaptive myocardial atrophy occurs, further dampening cardiac reserve [6]. With SCI, these conditions are exacerbated during upper extremity exercise such that with increasing workloads, whole-body peripheral resistance decreases (circulatory hypokinesis) and cerebral perfusion is diminished resulting in pre-syncopal or syncopal events [14,15,16].

Autonomic dysreflexia (AD) is defined as acute hypertension generated from uninhibited sympathetic reflexes resulting in baroreceptor mediated bradycardia [17]. AD was first described by Bowlby in 1890 in an 18-year-old man 6 months after what is described as C7 tetraplegia while passing a urinary catheter causing “a tingling and pricking sensation over the chest … profuse perspiration over the head, face and neck and the development of a bright red rash which persists for fifteen or 20 min.” [18]. In fact, any noxious stimuli below the SCI will increase excitatory post-synaptic potentials (EPSPs) at the preganglionic sympathetic neurons that are no longer opposed by supraspinal inhibition, especially in patients injured at T6 or above. The greater splanchnic nerve usually arises from T7 and T8 and innervates the entire splanchnic arterial bed; loss of supraspinal inhibition in SCI leads to unopposed vasoconstriction of this large vascular bed as well as the lower extremities in response to noxious input from below [5,17,19,20]. There are limited cases of AD in patients with T8–12 attributed to anatomic anomalies of the greater splanchnic nerve. Similarly, the nerves to the adrenal medulla typically arise from T7–T8, and when EPSPs are uninhibited by supraspinal influence due to SCI, activate secretion of epinephrine from the adrenal medulla directly into the blood stream which further exacerbates the hypertensive crisis. Of note, sympathetic blunting reduces circulating catecholamines, leading to increased numbers and sensitivity of adrenergic receptors (upregulation), further amplifying the reflex sympathetic activity [1,6]. Insults are often bladder distension, bowel obstruction and pressure injuries, but they can be precipitated from other etiologies including an ingrown toenail, lower limb fractures or sprains, deep venous thrombosis, spasticity, orgasm, and GI or GU instrumentation [5,17,19,20]. Sympathetic hyper-responsiveness may also manifest as pupillary dilation (blurred vision), upper body sweating, cutis anserina and parasympathetically-mediated symptoms of headaches, flushing above the SCI and nasal congestion; some individuals display none of the signs or symptoms listed above [2,5,19]. AD is clinically manifested as an increase in 20 mmHg systolic blood pressure (SBP) above baseline [2,5]. Persistent AD, in which the SBP may exceed 300 mmHg, can result in life threatening complications such as cerebrovascular accidents, myocardial infarction, hypertensive encephalopathy, retinal detachment, seizures and pulmonary edema [2,5,17,19,20]. Incidence increases with completeness of injury; 92% of patients with SCI above T6 will experience episodes of AD within the first year [2,5]

### 5.2. Thermoregulatory Dysfunction

Individuals with SCI are often described as poikilothermic, i.e., taking on the ambient temperature around them due to the inability to regulate core body temperature. Thermoregulation in humans is normally coordinated by the thalamus and the preoptic area of the hypothalamus (POAH) after receiving afferent signals from thermal receptors in the periphery. The POAH modulates thermoregulation by optimizing respiratory convective and evaporative heat exchange, as well as radiation, convection, conduction evaporation of heat exchange from the skin surface [21]. During periods of excess body temperature, warm blood in the body’s core is shunted to the periphery where cutaneous vasodilation promotes cooling at the surface of the skin through the mechanisms described above. Unfortunately, after high thoracic and cervical SCI, sympathetic blunting prevents such shunting and further inhibits sweating below the level of injury; above the SCI, skin may be flushed, and excess sweating may occur as the body tries to dissipate heat [22,23,24]. Neurogenic fever (Temperature > 37.7 °C) is likely to occur as the core temperature increases, and theories have included disconnection of the POAH with spinal cord sensory information, acute increases in neurotransmitter release, free radical production, and blood within the intraventricular space. Most likely, however, is the simple inability to use countercurrent exchange to dissipate heat from the body core to skin surface due to sympathetic blunting [20,25]. Neurogenic fever is a diagnosis of exclusion, such that other etiologies including infection, deep venous thrombosis, pulmonary embolism, heterotopic ossification, and drug fevers must be ruled out [26,27]. Treatment of neurogenic fever involves altering posture, thermal environmental regulation including clothing changes, and trials of antipyretics, bromocriptine, amantadine, dantrolene, and propranolol have been attempted with variable success [26]. Notably, exercise-induced hyperthermia is common in persons with SCI who cannot sweat below the level of injury; excess sweating above the SCI has very limited ability to dissipate heat through evaporation. Additionally, of note, excess sweating (hyperhidrosis) is often associated with AD, as the reflex sympathetic outflow activates cholinergic sweat glands [2,5]. Conversely, in cold environments, the countercurrent exchange (core-to-surface, surface-to-core) is also impaired, and core temperatures may drop precipitously, resulting in hypothermia for persons with high thoracic and cervical SCI [28,29].

### 5.3. Respiratory

SNS blunting due to cervical and high thoracic SCI has profound influence on respiratory physiology acutely, and although mitigated in chronic SCI by progressive partial PNS withdrawal over time, parasympathetic dominance and blunted supraspinal sympathetic drive continues to dampen “crisis” responses chronically. Unopposed vagal influence on the bronchial tree results in bronchiolar constriction, hyper-reactive airways and increased mucus secretion [30]. Coupled with neurogenic restrictive lung disease, a result of intercostal and abdominal muscle paralysis, individuals with cervical/high thoracic SCI are at high risk of atelectasis, pneumonia and mucus plugging, as they are unable to voluntarily cough/clear pulmonary secretions, even at rest [31]. Under exercise or “crisis” conditions, in addition to ventilatory insufficiency, inspiratory effort is limited by persistent bronchoconstriction and mucus production despite increased ventilatory requirements at higher workloads [32].

### 5.4. Genitourinary Dysfunction

Bladder storage and emptying is the result of carefully regulated supraspinal coordination of SNS, PNS and the somatic nervous system which are mediated through the hypogastric nerve (T10–L2), pelvic splanchnic nerves (S2–S4) and pudendal nerve (S2–S4), respectively [1]. SNS activity mediates bladder filling through activation of α_1_ adrenergic receptors at the bladder neck and β_3_ adrenergic receptors on the detrusor dome causing relaxation of the body of the detrusor. Conversely, PNS activity mediates bladder emptying primarily due to its effect on detrusor M_3_ cholinergic receptors. After SCI, both systems are subject to increased uninhibited activation by unmyelinated Aδ stretch and C pain receptors as the detrusor fills, resulting in reflex detrusor contraction opposed by bladder neck contraction [2,33]. At the same time, muscle spindles in the external urethral sphincter are activated to cause reflex sphincter/pelvic floor contractions mediated by the pudendal nerve. The net result is detrusor sphincter dyssynergia generating high bladder pressures, and probable vesicoureteral reflux associated with hydroureter, hydronephrosis and urinary incontinence [2,33]. Of note, urinary dysfunction, including bladder distension, stones, urinary tract infections, detrusor sphincter dyssynergia, trauma, shock wave lithotripsy and urological instrumentation, is the most likely etiology of acute onset AD.

Sexual responses are also under a coordinated combination of SNS, PNS and somatic control. Persons with SCI above L2 may likely have preserved psychogenic arousal that requires an intact hypogastric nerve (T11–L2) and intact SNS. An intact pelvic nerve (S2–S5) is necessary to mediate parasympathetic reflexogenic arousal, although genital sensation requires intact innervation down to and through the pudendal nerve (S2–S4). Disruption of the SNS, PNS and somatic nervous system following SCI often results in erectile dysfunction, diminished lubrication, retrograde ejaculation, infertility, and abnormal semen quality in men [34], with commensurate dysfunction in women, although fertility is usually preserved [35,36]. As with urinary dysfunction, genital etiologies of AD are frequent, including orgasm, priapism, epididymitis, scrotal compression, trauma, penile vibratory stimulation, electroejaculation, menstruation, pregnancy, labor, delivery, and breast feeding [5,34].

### 5.5. Gastrointestinal Dysfunction

As mentioned above, the ENS can function independently to digest and eliminate foodstuffs, although it is usually under SNS, PNS and somatic nervous system influences. Because of sympathetic blunting and parasympathetic dominance, gastric acid secretions may be increased acutely and chronically after high thoracic and cervical SCI [37]. Similarly, biliary sludge, cholelithiasis and cholecystitis rates have been demonstrated to be higher in persons with SCI at these levels as a result of vagal-mediated parasympathetic influence [38,39]. Conversely, relatively absent parasympathetic influence due to SCI may contribute to increased transit time at the distal colon that is innervated by the pelvic splanchnic nerves (S2–S4) [1]. Increased uninhibited activation of pelvic splanchnic nerves by unmyelinated Aδ stretch and C pain receptors associated with colon and rectal vault expansion results in reflex colorectal contractions opposed by reflex somatic external sphincter, pelvic floor and puborectalis contractions mediated by the pudendal nerve in response to muscle spindle activation. Concomitant constipation and bowel incontinence often results. Of note, bowel dysfunction, including cholelithiasis, gastric ulcers, constipation, obstruction, acute abdomen, hernia, hemorrhoids, cancer, trauma, and GI instrumentation, is the second most likely etiology of acute onset AD.

### 5.6. Diagnosing Autonomic Dysfunction

Initial autonomic assessment should include the International Standards to determine remaining Autonomic Function after Spinal Cord injury (ISAFSCI) [2] used in concert with the International Standards for the Neurological Classification of SCI (ISNCSCI) [40]. This tool documents cardiovascular, bronchopulmonary and sudomotor symptomatology as well as genitourinary, bowel and sexual function providing a clinical “score” of autonomic dysfunction [2]. Additional objective testing might include non-invasive sympathetic skin sweat responses (SSR) to electrophysiologic stimulation of median and tibial nerves to quantify risk of autonomic dysreflexia. In a study by Curt et al., SSR significantly corresponded to the neurologic level injury by ISNCSCI exam in complete SCI and predicted the likelihood of developing chronic autonomic dysreflexia; however, SSR did not show significant predictive value in those patients with incomplete lesions [41].

## 6. Management of Autonomic Dysfunction after SCI

Following acute recovery of neurogenic shock, autonomic dysfunction may persist due to loss of supraspinal influence, SNS blunting, PNS dominance and unchecked reflex sympathetic outflow in response to noxious stimuli below the level of injury. Long term complications of these alterations include cardiac dysrhythmias, neurogenic orthostatic hypotension, autonomic dysreflexia, thermal dysregulation, immune system dysregulation, neurogenic bowel, neurogenic bladder, as well sexual dysfunction, and infertility.

### 6.1. Cardiovascular Management

Cardiac dysrhythmias due to uncompensated sympathetic blunting and parasympathetic dominance may include bradycardia, supraventricular tachycardia, sinus nodal arrest or cardiac arrest especially during the first month [6,20]. Management, especially in the initial hospitalization, should rely on telemetry monitoring and subsequent ACLS protocols [6,20]. Cardiology consultation is warranted for persistent dysrhythmias beyond the period of spinal shock, and pharmacological intervention or pacemaker placement may be necessary.

Initial NOH should be systematically addressed using mechanical and nonpharmacological strategies before considering pharmacological intervention. NOH symptoms may include fatigue, weakness, light-headedness, dizziness, blurred vision, dyspnea, and restlessness. Accurate diagnosis of NOH with sit-up test or head-up tilt test is essential to establish baseline blood pressure responses. Adequate hydration with fluids and sodium intake to expand plasma volume should be trialed, although evidence to support these interventions is weak. Mechanical interventions including abdominal binders, and, to a lesser degree, lower extremity compression stockings may facilitate venous return and improve myocardial contractility, improving MAP during seated activities [5,12,20]. Progressive tilt table elevation may improve NOH tolerance over time, and power wheelchairs with tilt-capacity may be warranted in both acute and chronic settings. If NOH persists despite these measures, pharmacological intervention should be considered. While midodrine, a peripheral α1-agonist, has strong evidence for NOH efficacy in non-SCI populations [12], only weak evidence for supporting its use is available for those with SCI-induced NOH [5,42]. Nonetheless, it is considered first line pharmacotherapy for NOH after SCI. Additional medications for consideration, used alone or in concert with midodrine, include fludrocortisone, a synthetic mineralocorticoid, and droxidopa, a precursor to norepinephrine; both have weak evidence for improving NOH in SCI [5,12]. Blood pressure and symptoms should be continually assessed as one or more of these medications are introduced and titrated upward; the new baseline BP and heartrate should be documented in the medical record.

### 6.2. Managing Autonomic Dysreflexia

**Figure 3:**
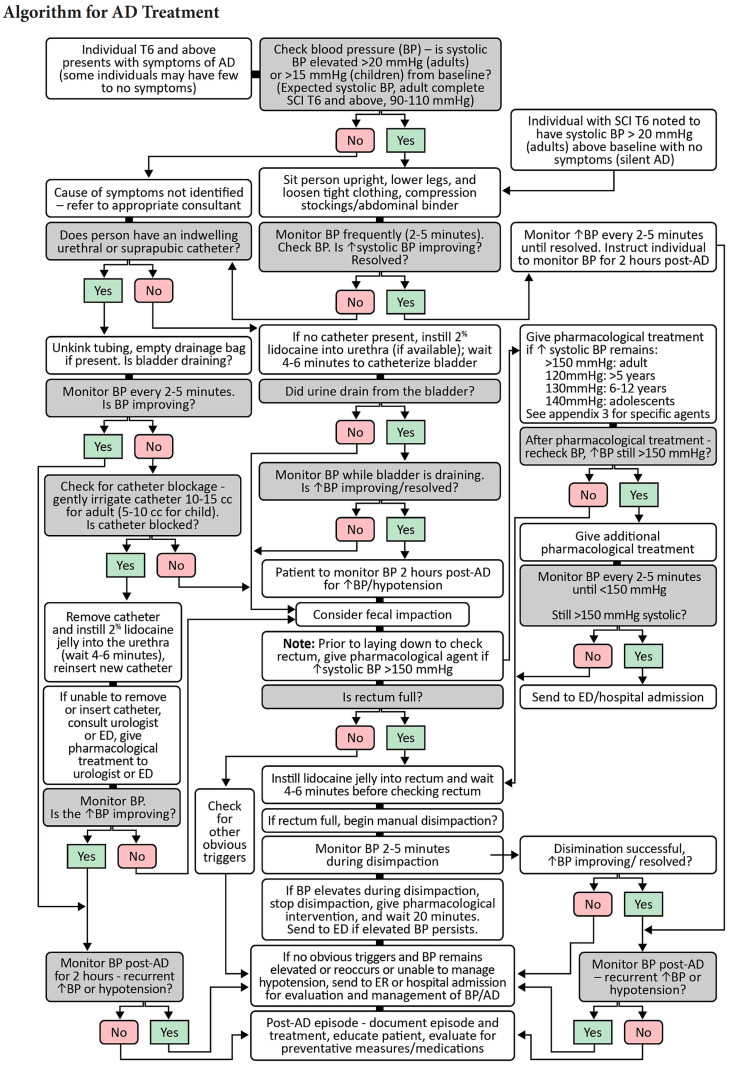
Management algorithm for Autonomic Dysreflexia. Reprinted with permission from Topics in Spinal Cord Injury Rehabilitation, Evaluation and Management of Autonomic Dysreflexia and Other Autonomic Dysfunctions: Preventing the Highs and Lows: Management of Blood Pressure, Sweating, and Temperature Dysfunction. Krassioukov et al. TSCIR 2021;27(2):225–290. Copyright 2021 by American Spinal Injury Association.

With pseudonyms of autonomic hyperreflexia, mass reflex, paroxysmal hypertension, sympathetic hyperreflexia, paroxysmal neurogenic hypertension and autonomic spasticity, autonomic dysreflexia (AD) is a potentially life-threatening condition that can occur in anyone with an SCI at or above T6 in response to a noxious stimulus below the level of injury. As such, it is imperative to remove the noxious stimuli and rapidly manage the systemic hypertension (Figure 3). Blood pressure should be monitored every 2–5 min via sphygmomanometer until AD has resolved. Begin by sitting patient upright and removing restrictive clothing, including abdominal binders and compression garments. If elevated blood pressure persists, consider unkinking tubing of an indwelling catheter, emptying the drainage bag and ensuring bladder is draining. Check for indwelling catheter blockage by gently irrigating with 10 cc normal saline at body temperature; if blocked, remove and replace the catheter, after first instilling 2% lidocaine into the urethra. A coude tip catheter may be required if there is a history of urethral scarring, false passage, or strictures. Emergent Urology consultation may be warranted if unsuccessful. If there is no indwelling catheter, instill 2% lidocaine into the urethra, followed in 4–6 min by catheterizing the potentially distended bladder; a coude tip catheter may be required as above. Consider pharmacologic intervention if SBP remains ≥ 150 mm Hg, before lying the person down to check for fecal impaction. Instill 2% lidocaine jelly into the rectum and wait 4–6 min before performing a digital rectal examination. If rectum is full of stool, gently proceed with manual disimpaction; discontinue if AD becomes worse or stool cannot be removed. If SBP remains > 150 mmHg, consider additional pharmacological intervention/topical anesthetic and wait 20 min before proceeding with aggressive bowel evacuation. If bladder or bowel are not implicated, consider additional triggers described above, including potential myocardial infarction and initial evaluation with echocardiogram or troponin labs. Identification and removal of the noxious stimuli remains of paramount importance, although worsening hypertension must be managed pharmacologically until etiology is found. Acute pharmacologic intervention begins with the application of 1–2 inches of 2% nitroglycerin gel above the level of lesion and removed when blood pressure and symptoms resolve; note this agent is contraindicated within 24 h of using sildenafil/vardenafil, or within 48 h of using tadalafil [5]. A second line agent includes oral administration of nifedipine 10 mg capsules with immediate release formulation; sublingual administration is not recommended because of its variable and unpredictable absorption [5]. Nifedipine should also be avoided in elderly individuals, those with liver disease and persons with acute angina. Other alternatives include captopril, clonidine, and hydralazine in the acute setting. Phenoxybenzamine, an α_1_ and α_2_ antagonist, clonidine, an α_2_ antagonist, and other alpha-adrenergic antagonists may be efficacious in managing recurrent AD due to an ongoing noxious stimuli such as fracture, pressure injury or renal stone, until those resolve [43].

There are special considerations with AD interventions, including child and adolescent management, outpatient gastroenterological and urologic procedures, pregnancy, sexual function, and athletes with high thoracic and cervical SCI who intentionally invoke AD as an ergogenic aid to offset circulatory hypokinesis during competition.

A child’s blood pressure threshold will increase as the patient ages; AD intervention should begin when systolic blood pressure is greater than 120 mmHg before age 5, 130 mmHg before age 12, and 140 mmHg in adolescents [5]. Proper care should be taken to evaluate blood pressure with the appropriately sized sphygmomanometer, as improper use can distort values.

For persons with SCI undergoing outpatient procedures, there are ongoing studies of appropriate prophylactic pharmacologic management and timing. GI prep for anorectal procedures and colonoscopy can precipitate AD, even when modified administration is provided over several days, and inpatient monitoring for those persons at risk is warranted [44]. In a recent randomized, placebo-controlled trial, lidocaine anal block was demonstrated to significantly reduce AD during anorectal procedures, including flexible sigmoidoscopy and/or anoscopic hemorrhoid ligation [45].

Similarly, urological procedures including cystoscopy and urodynamic studies can precipitate AD, and SBP monitoring every 2 min is advised during and immediately after the procedure [5,17,33]. For individuals prone to AD, lidocaine jelly instillation into the urethra 3–5 min prior to urethral instrumentation should be considered. AD prophylaxis with an alpha-blocker the night before cystoscopic procedures and/or an anticholinergic medication within 1 h of the procedure should be considered for individuals at high risk [5]. While AD prophylaxis is not recommended for urodynamics because an important aspect of the study is to observe BP responses to bladder filling and voiding, pharmacologic agents should be available at bedside during the procedure in case they are urgently needed to treat AD [5]. In both men and women with high thoracic and cervical SCI, there is a risk of AD with sexual stimulation and vibrostimulation; individuals should be encouraged to monitor and stop activity if AD begins. Of note, nitrates are contraindicated for those using phosphodiesterase 5 inhibitors and develop AD associated with priapism or sexual activity. An outpatient clinic trial with penile vibratory stimulation (PVS) is recommended prior to home use to determine relative risk and management of AD. During electroejaculation or other sperm retrieval procedures, the specialist administering anesthesia should be monitoring BP, although spinal or epidural anesthesia should block the afferent stimuli that could potentially cause AD [5,34].

For women with high thoracic and cervical SCI of childbearing age, pregnancy should be considered as a possible stimulus for AD episodes. Pregnancy, labor, delivery, and breastfeeding have shown to be potential risks for developing new onset AD, and a high-risk multidisciplinary team is recommended throughout [36,46,47,48]. Education of risks and symptoms for both patients and providers is recommended. Providers should be advised that AD can mimic pre-eclampsia, and both conditions should be carefully considered and managed throughout the pregnancy [5,36,46,47,48]. Antepartum consultation with the anesthesiologist should be provided, as well as a plan for epidural/spinal anesthesia to mitigate AD during labor, delivery and initial post-partum breastfeeding [5,36].

“Boosting” is the intentional induction of AD by athletes with high thoracic and cervical SCI as an ergogenic aid to offset circulatory hypokinesis during competition [49]. In eight athletes who practiced boosting, Wheeler et al. reported norepinephrine levels at rest 220% higher in boosted versus unboosted state, while post-exercise norepinephrine levels were 295% higher in the boosted versus unboosted condition [50]. The same group had previously reported 9.7% faster race times and increased VO_2Peak_ in the boosted state for that same cohort [51]. The International Paralympic committee has declared boosting as illegal and has taken steps to curtail the practice in paralympic athletes due to the serious risks and complications associated with its practice. If SBP is > 160 mmHg within 30 min of an event, the athlete is to be re-examined 10 min later; a second SBP > 160 mmHg would disqualify the athlete from the event in question [5].

### 6.3. Thermodysregulation

As noted above, persons with high thoracic and cervical SCI are considered poikilothermic and are especially susceptible to hyperthermia or hypothermia, under conditions of extreme heat/humidity or extreme cold, respectively [2,5]. Core temperature should be monitored during exercise in hot environments, and individuals with SCI may require air conditioning, cooling vests, cold towels, cool liquids and rest to treat neurogenic hyperthermia. Conversely, neurogenic hypothermia in persons with SCI may require warming blankets, warm humidified air, and consumption of warm fluids [5].

### 6.4. Managing Hyperhidrosis

Hyperhidrosis appears to be an exaggerated reflex sympathetic response to noxious stimuli below the level of injury in persons with high thoracic and cervical SCI. It may occur separately or coincide with other signs and symptoms of AD, and therefore, an early imperative is to locate and remove the noxious stimuli as primary intervention. Its prevalence as determined by a 1992 questionnaire survey of persons with SCI in Denmark is ~27% [52], and management strategies have included analgesics (including dextropropoxyphene [52], gabapentin [53]. and indomethacin [54]), anticholinergic agents [55,56], α adrenergic antagonists [57,58,59], and sympathotomy [60]. Botulinum toxin injection may be another potential treatment option for site specific sweating, as it has been successfully used in the management of primary hyperhidrosis [61].

### 6.5. Managing Genitourinary and Gastrointestinal Dysfunction

Neurogenic bladder, sexual dysfunction and neurogenic bowel management strategies are beyond the scope of this manuscript and will be discussed separately in this special issue.

## 7. Conclusions

Autonomic dysfunction is among the most profound changes following SCI, yet it is often overlooked due to society’s focus on paralysis and inability to walk. Nonetheless, a national survey conducted by KD Anderson in 2004 demonstrated that persons with tetraplegia and paraplegia report the elimination of autonomic dysfunction and recovery of bowel, bladder, and sexual function as a more desirable return of function than the ability to walk. The survey provides perspective into the importance of developing strategies to manage various autonomic dysfunction in the setting of SCI. Sympathetic blunting and dissociation from supraspinal controls in the unique setting of ANS parasympathetic dominance contributes to neurogenic bradycardia, neurogenic orthostatic hypotension, adaptive myocardial atrophy, thermoregulatory dysfunction, neurogenic obstructive lung disease/airway hyper-reactivity, neurogenic bowel, neurogenic bladder, and sexual dysfunction. Additionally, uninhibited and exaggerated reflex sympathetic outflow in response to noxious stimuli below the level of injury for those with high thoracic and cervical SCI can cause embarrassing hyperhidrosis and life-threatening hypertensive crisis. Managing these autonomic disturbances should therefore be considered of paramount importance when providing clinical care to this vulnerable population.

## Data Availability

Not applicable.

## Abbreviations

ANS	autonomic nervous system
AD	autonomic dysreflexia
ISAFSCI	International Standards to Determine Remaining Autonomic Function after Spinal Cord Injury
INCSCI	International Standards for Neurological Classification of Spinal Cord Injury
NOH	neurogenic orthostatic hypotension
PNS	parasympathetic nervous system
SCI	spinal cord injury
SNS	sympathetic nervous system
SSR	sympathetic skin response
